# The Pocket Anatomist

**Published:** 1861-01

**Authors:** 


The Pocket Anatomist; being a complete description of the Anatomy of the human body, for the use of Students. By M. W. Hilles, formerly Lecturer on Anatomy and Physiology at the Westminister Hospital School of Medicine, &c. Philadelphia; Lindsay and Blakiston. 1860.

This is a small duodecimo volume of 263 closely printed pages, designed chiefly as an aid to the student in preparing for his examinations.

For Sale by S. C. Griggs & Co., Chicago.

